# Synthesis of Ag@Silica Nanoparticles by Assisted Laser Ablation

**DOI:** 10.1186/s11671-015-1105-y

**Published:** 2015-10-13

**Authors:** JR González-Castillo, E. Rodriguez, E. Jimenez-Villar, D. Rodríguez, I. Salomon-García, Gilberto F. de Sá, T. García-Fernández, DB Almeida, CL Cesar, R. Johnes, Juana C. Ibarra

**Affiliations:** Instituto Politécnico Nacional, CICATA U. Altamira, Altamira, CP 89600 México; Departamento de Investigación Aplicada, Driscoll’s-México, Jalisco, CP 45050 México; Universidade Federal de Pernambuco, DQF, Recife, CP 50670-901 Brazil; Universidad Autónoma de la Ciudad de México, México DF, CP 09790 México; Universidade Estadual de Campinas, IFGW-DEQ, Campinas, CP 13083-859 Brazil

**Keywords:** Silver nanoparticles, Core-shell nanoparticles, Assisted laser ablation, Surface plasmon resonance

## Abstract

This paper reports the synthesis of silver nanoparticles coated with porous silica (Ag@Silica NPs) using an assisted laser ablation method. This method is a chemical synthesis where one of the reagents (the reducer agent) is introduced in nanometer form by laser ablation of a solid target submerged in an aqueous solution. In a first step, a silicon wafer immersed in water solution was laser ablated for several minutes. Subsequently, an AgNO_3_ aliquot was added to the aqueous solution. The redox reaction between the silver ions and ablation products leads to a colloidal suspension of core-shell Ag@Silica NPs. The influence of the laser pulse energy, laser wavelength, ablation time, and Ag^+^ concentration on the size and optical properties of the Ag@Silica NPs was investigated. Furthermore, the colloidal suspensions were studied by UV–VIS-NIR spectroscopy, X-Ray diffraction, and high-resolution transmission electron microscopy (HRTEM).

## Background

Nowadays applications of metallic nanoparticles (NPs) in different areas of science and technology are increasing. In recent years, researchers have focused their efforts to synthesize these materials and to study their optical properties, which strongly depend on the size and shape of the NPs [1]. An important part of these studies has been focused on the surface plasmon resonance (SPR), which consists in a resonance coupling between the electromagnetic field frequency and the free electrons oscillation frequency on the metal NPs. When resonance conditions are matched, a strong absorption of the electromagnetic field energy is brought about by metallic NPs [2]. Thanks to these properties, Ag NPs have been widely used as biological labeling [3] and as antibacterial agents [4–7], among others [8, 9]. Different physical and chemical methods have been used to obtain NPs [10–15], among them is the laser ablation in liquid [16, 17].

Here, by using a new variant of laser ablation technique (assisted laser ablation) core-shell Ag@Silica NPs were synthesized [18–20]. Among other advantages, the silica coating provides inertness [4, 21, 22] and high dispersibility of the NPs [23, 24] which has enabled their use in numerous applications [25, 26]. In these previous reports, the silver salt was added before the target ablation, which led to the continuous production of silver NPs during the laser ablation process. This fact has the important disadvantage of the extinction of the laser radiation produced by the silver NPs. This effect provokes a continuous reduction of the laser intensity at the target surface, which in turn prevents the obtaining of higher NPs concentrations. In this paper, the silver salt is introduced immediately after the irradiation of the silicon target, producing also Ag@Silica NPs. Furthermore this paper is aimed to get a deeper insight in the influence of the growing parameters on the morphological, optical and structural properties of the NPs.

## Methods

**Fig. 1:**
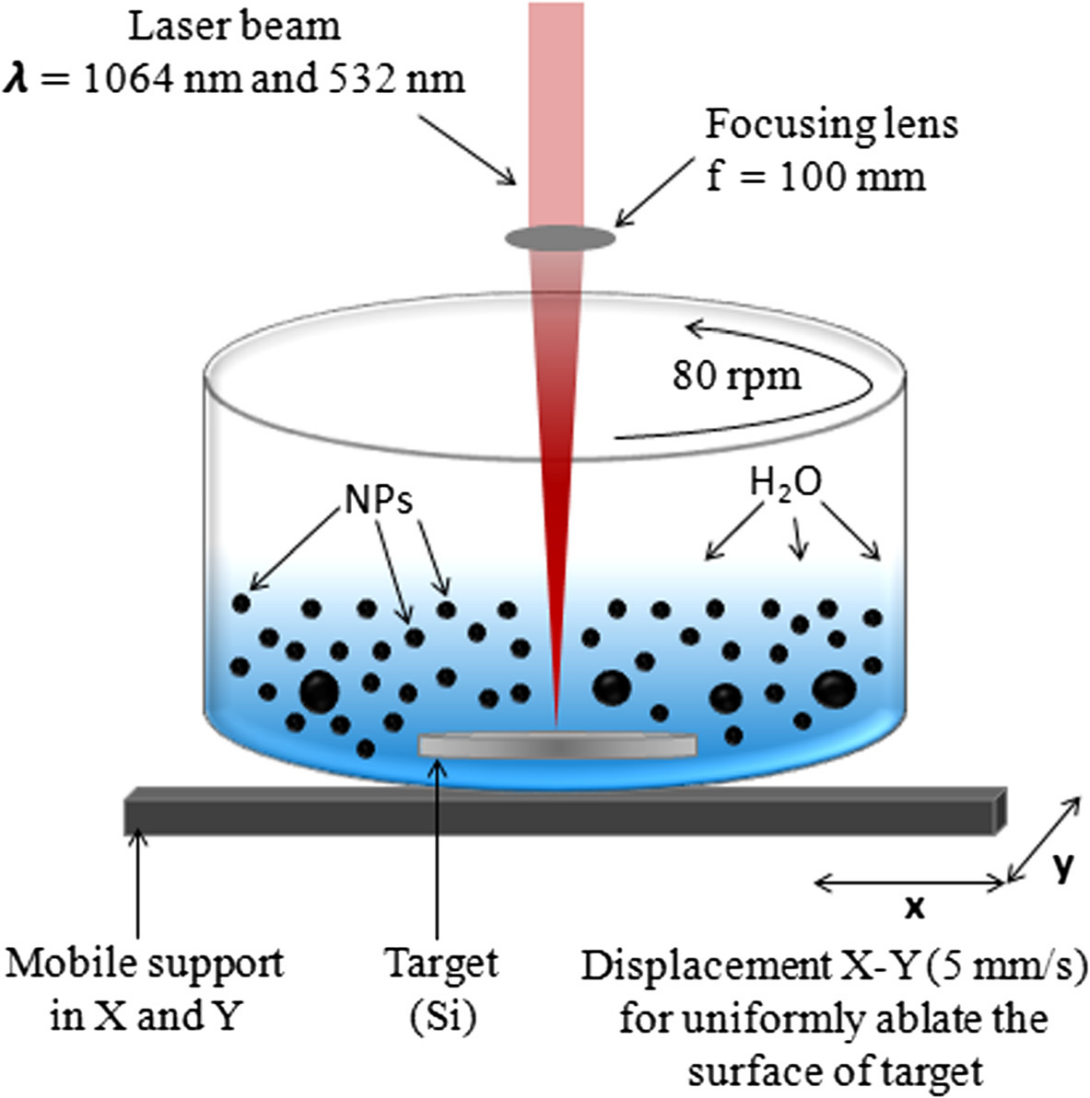
Schematic diagram of the experimental set up for NPs production

Figure 1 shows a schematic representation of the experimental set up used for the synthesis of Ag NPs coated with silica (Ag@Silica NPs) using an assisted laser ablation technique. It is basically a redox chemical synthesis, where one of the reactants (silicon) is supplied in nanometer dimensions by pulsed laser ablation and then a [AgNO_3_] is added to the solution. This technique involves focusing laser pulses on a 99.99 % pure silicon target placed at the bottom of a beaker containing 20 mL of deionized water. In order to remove any contaminant that could be present at the target surface before the ablation process, the silicon target was successively cleaned in acetone, isopropanol, and deionized water for 5 min each in an ultrasonic bath. The silicon target was ablated using a *Q*-switched Nd:YAG laser (Quantel Brio), which delivers 5 ns laser pulses at 20 Hz. Samples were prepared using both the fundamental (1064 nm) and second harmonic (532 nm) of the laser radiation.

Optical and structural properties of the Ag@Silica NPs were studied as functions of the laser pulse energy (from 5 to 50 mJ), ablation time (from 1 to 25 min), [AgNO_3_] (from 5 × 10^−5^ to 5 × 10^−4^ M), and laser wavelength (1064 and 532 nm). The resulting colloids were examined by high-resolution transmission electron microscopy (HRTEM) on a JEOL JEM3010 300 kV with a thermionic electrons source. Structural properties of the samples were also studied by X-Ray diffraction (XRD) using a Bruker XRD D8 Advance diffractometer in Bragg Brentano configuration for powders and a sweeping from 20° to 80°. The samples for the XRD study were prepared by drop casting of the Ag@Silica NPs colloids on a hot glass plate (50 °C), in order to evaporate the H_2_O content and to form a film thick enough to be measured by XRD. The absorption spectra of the colloidal suspensions were measured using a Perkin-Elmer Lambda 9 UV–VIS spectrophotometer and a quartz cuvette with 10 mm of path length.

## Results and Discussion

### Influence of the Laser Energy on the NPs Properties

For this study, an ablation time of 20 min, a wavelength of 532 nm, and a [AgNO_3_] of 1.25 × 10^−4^ M were used. Every colloid was obtained using a constant laser pulse energy, which took values between 5 and 50 mJ.

**Fig. 2:**
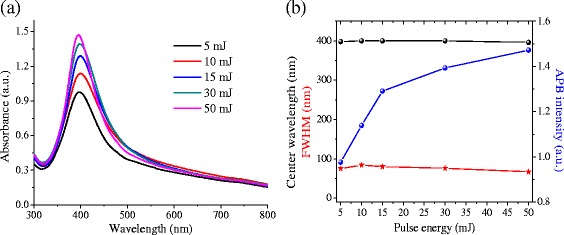
**a** Absorption spectra of colloidal suspensions synthesized with *λ* = 532 nm, *f* = 20 Hz, *t* = 20 min, (1.25 × 10–4 M) of AgNO3, and laser pulse energy between 5 and 50 mJ. **b** FWHM, center wavelength, and intensity of APB vs. laser pulse energy

Figure 2a shows absorbance spectra of the colloidal suspensions prepared using the different laser pulse energies, and Fig. 2b presents the dependence of the basic features of the absorption plasmon band (APB) namely its center wavelength (CWL), full width at half maximum (FWHM), and maximum intensity on the laser pulse energy. According to the results, both the CWL and the FWHM exhibit no significant dependence on the laser pulse energy, with variations of less than 0.5 and 11.2 %, respectively. These results suggest that both the size and size distribution of the NPs are similar within the studied pulse energy interval. However, the APB intensity increases more than 40 % when the laser pulse energy is increased from 5 to 50 mJ, suggesting an increase in the [NPs] as the laser energy is increased. For energy ≥45 mJ, the APB intensity exhibits a trend to saturation, presumably due to the total reduction of Ag^+^ ions.

**Fig. 3:**
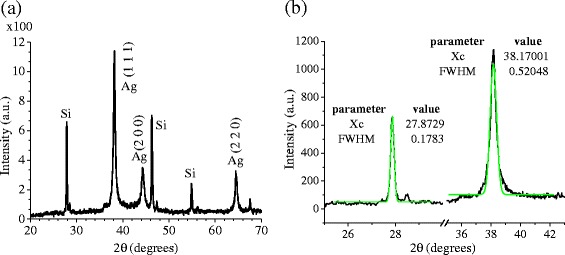
**a** XRD pattern resulting from the characterization of a film produced from colloidal samples containing Ag@Silica NPs and **b** zoom of the same pattern, which shows fitting curves used to determine de parameters of the peaks. The samples were prepared using a laser pulse energy of 15 mJ, *λ* = 532 nm, *f* = 20 Hz, *t* = 20 min, and (1.25 × 10–4 M) of AgNO3

Figure 3 shows a typical XRD spectrum of the synthesized samples. The diffractogram shows peaks at 2θ = 38.1°, 44.3°, and 64.5°, corresponding to the reflections of the silver crystalline planes (111), (200), and (220), respectively. According to the 4–0783 JCPDS card, those peaks belong to the FCC silver structure. The calculated lattice parameter *a* = 0.408 nm, as well as the interplanar distances *d*_111_ = 0.236 nm, *d*_200_ = 0.204 nm, and *d*_220_ = 0.144 nm agree well with the reported ones. Using these reflections, the Scherrer formula, and a Gaussian distribution for the diffracted radiation intensity, it was possible to estimate the size *D*_Ag_ of the silver NPs in the different crystalline directions: *D*_111_ = (16.2 ± 0.2) nm, *D*_200_ = (13.0 ± 0.2) nm, and *D*_220_ = (17.4 ± 0.3) nm. Notice that the NP size, estimated by the Scherrer formula, is different for the planes (111) and (200), which would mean the non sphericity of the NPs. The average crystallite size taken over all crystalline directions was ${D}_{\mathrm{Ag}}^{\mathrm{ave}}=\left(16.0\pm 0.1\right)\ \mathrm{nm}.$

In order to obtain a more accurate value of the average diameter of the NPs, we used the modified Scherrer formula suggested by Ahmad et al. [27]. The well-known Scherrer formula can be rewritten as:

$$ \beta =\frac{K\ \lambda }{D \cos \left(\theta \right)} $$

where *K* ≈ 0.89, *λ* = 1.5406 Å, *β* is the FWHM of the peak, *D*_Ag_ is the NPs diameter, and *θ* is the central angle of the diffraction peak. Taking the natural logarithm on both sides of the formula, the following equation is obtained:

$$ \ln\ \left(\beta \right)= \ln\ \left(\frac{K\ \lambda }{D}\right)+ \ln\ \left(\frac{1}{ \cos \left(\theta \right)}\right) $$

By plotting ln (*β*) along the y-axis vs. $\ln\ \left(\frac{1}{ \cos\ \left(\theta \right)}\right)$ along the x-axis, for all the considered peaks (*θ*_*i*_,*β*_*i*_), the result should approximate to a straight line with a slope = 1 and an intercept $p= \ln \left(\frac{k\ \lambda }{D_{\mathrm{Ag}}}\right)$. After a linear fitting of the experimental data using the least square method, the best-fit parameter *p* was calculated (*p* = −4.6 ± 0.3). Using this value, *D*_Ag_ can be obtained as:

$$ {D}_{Ag} = \left(\frac{k\ \lambda }{e^p}\right)=\left(14\pm 3\right)\ \mathrm{nm} $$

The diffractogram also reveals the presence of crystalline silicon with peaks located at 2θ = 27.8°, 46.2°, and 54.8°. The peak located at 2θ = 27.8° was used to estimate the size of the crystalline silicon present in the sample, *D*_Si_ = (46 ± 1) nm. This suggests that silicon particulates were ejected from the target by the shock waves produced during the ablation process. The presence of silicon crystals in the solution is also confirmed by the dispersion tail observed in the absorbance spectrum (Fig. 2a) between 500 and 800 nm. The error of the crystallite size was estimated by error propagation in the Scherrer formula, using the standard errors for FWHM and peak position obtained during the fitting process.

**Fig. 4:**
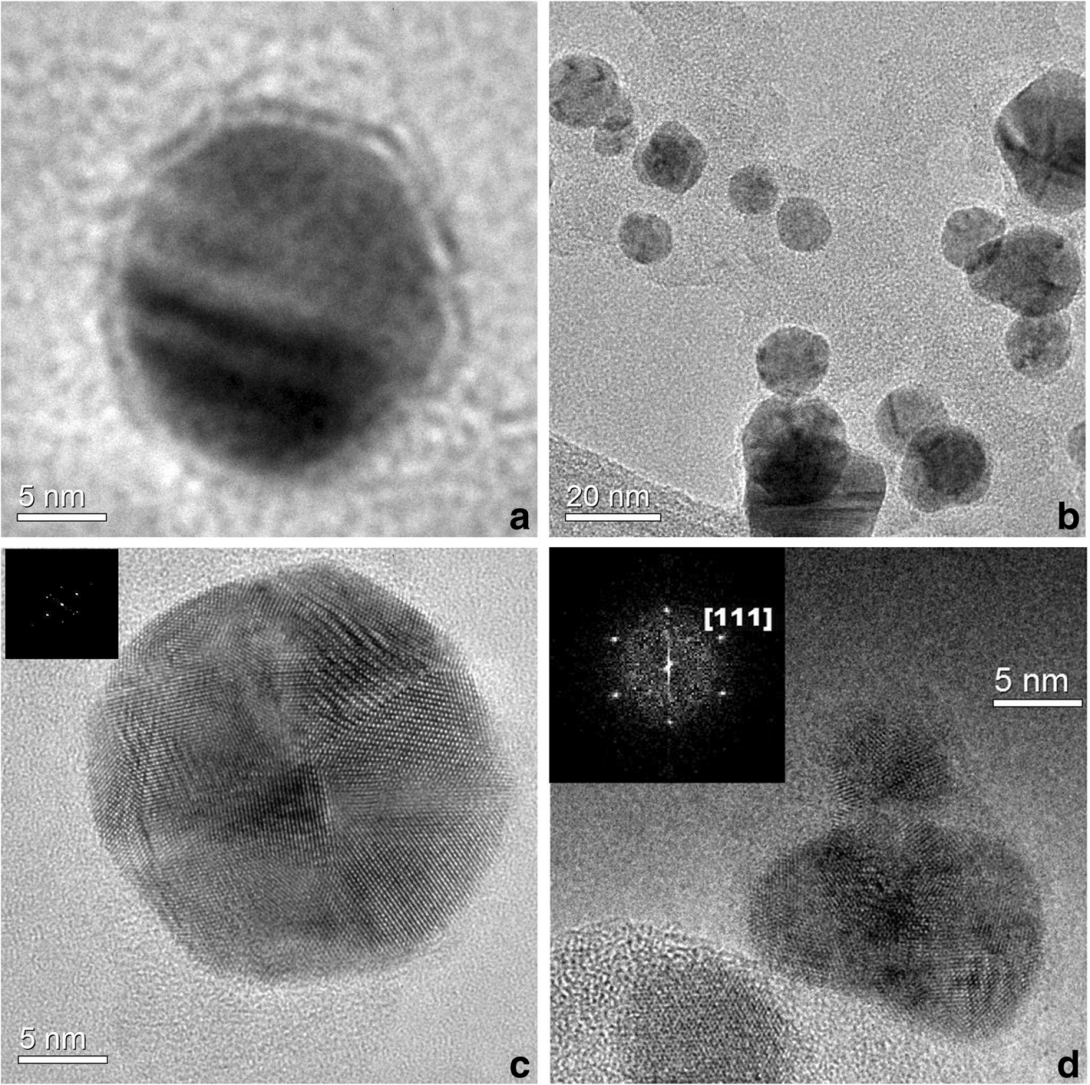
HRTEM images of Ag nanoparticles synthesized with *λ* = 532 nm, *f* = 20 Hz, *t* = 20 min, (1.25 × 10–4 M) of AgNO3, and using laser pulse energies of 15 mJ (**a**, **b**) and 50 mJ (**c**, **d**)

Figure 4 shows HRTEM images of the synthesized Ag@Silica NPs, revealing that Ag NPs are coated with an amorphous silica shell of (1–2 nm) thickness. The small thickness of the silica shell could be associated to the fast exothermic combustion reaction of Si NPs in aqueous solution, leading to their fragmentation and oxidation. HRTEM micrographs show that the silver NPs are crystalline in nature, typically characterized by multiple twinning planes. Figure 4c, d insets are the Fourier transform (FT) of Ag nanoparticles images, revealing the crystalline directions [111] and [200] of the Ag FCC structure. The interplanar distances, *d*_111_ = 0.236 nm and *d*_200_ = 0.204 nm, obtained from the FT analysis, are in agreement with the values obtained from the X-ray study.

The TEM images also reveal that there is no significant dependence of the nanoparticles size on the laser pulse energy. These results agree with results obtained by the absorbance measurements.

**Fig. 5:**
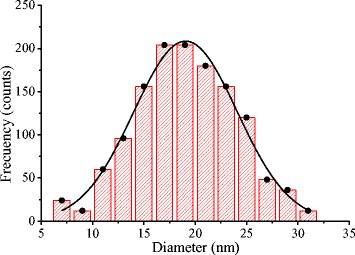
Size distribution of the Ag NPs synthesized using a laser pulse energy of 15 mJ, *λ* = 532 nm, *f* = 20 Hz, *t* = 20 min, and (1.25 × 10–4 M) of AgNO3

The HRTEM and TEM images were processed to obtain information on the size and size distribution of the Ag NPs. The size distribution study was carried out by manually outlining 1310 NPs from several dozens of low and high resolution TEM images. Once digitized and saved in the proper format, the images were processed using the Gatan Digital Micrograph program. Figure 5 shows a Gaussian distribution fitted to the histogram, with an average size ${D}_{\mathrm{Ag}}^{\mathrm{ave}}=\left(19.1\pm 0.2\right)$ and a standard deviation of *σ* = 5 nm.

### Influence of the Ablation Time on the NPs Properties

**Fig. 6:**
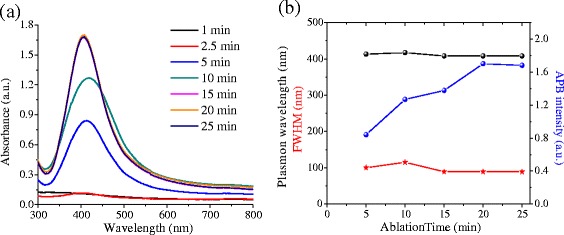
**a** Absorption spectra of colloidal suspensions (Ag@Silica NPs) synthesized using a laser pulse energy of 15 mJ, *λ* = 532 nm, *f* = 20 Hz, a [AgNO3] of 1.25 10–4 M and different ablation times. **b** CWL (*black*), FWHM (*red*), and APB intensity (*blue*) vs. ablation time

The absorbance spectra of colloidal suspensions prepared with different ablation times are presented in Fig. 6a. The wavelength used was *λ* = 532 nm. The results reveal that for low ablation times (1–2.5 min), APB is not observed. Further increase of the ablation time reveals the APB presence in the absorption spectra, showing a clear dependence of its intensity on the ablation time. Basic features of the APB are reported in Fig. 6b. For samples prepared with ablation times ranging from 5 to 25 min, the CWL exhibits a small variation (~1 %), and the FWHM presents a variation of 12.7 %, so it is inferred that the size and size distribution of nanoparticles do not exhibit a significant dependence on the ablation time. However, the APB intensity increases above 68 % with the increment of the ablation time, suggesting an increase of the [NPs]. For ablation times ≥15 min, the APB intensity exhibits a tendency to saturation, presumably due to the total reduction of Ag^+^ ions. This result suggests that despite more silicon is introduced into the solution, no more silver NPs are synthesized. This must be because the Ag^+^ has been exhausted.

### Influence of the [AgNO_3_] on the NPs Properties

**Fig. 7:**
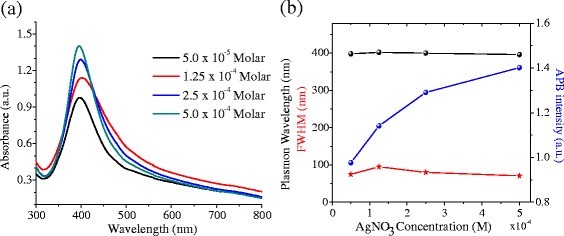
**a** Absorption spectra of the Ag@Silica NPs suspensions synthesized using a laser pulse energy of 15 mJ, *t* = 10 min, *f* = 20 Hz, *λ* = 532 nm, and different [AgNO3]. **b** Dependence of CWL (*black*), FWHM (*red*), and APB intensity (*blue*) on the [AgNO3]

Figure 7a shows absorbance spectra of the colloidal suspensions prepared for different [AgNO_3_]. The wavelength used was *λ* = 532 nm. For better appreciation of the APB parameters, Fig. 7b presents the dependence of the CWL, FWHM, and APB intensity on [AgNO_3_]. The CWL varies only 0.75 %, while the FWHM varies 14.4 % within the studied [AgNO_3_] interval, so it is inferred that the size and size distribution of the nanoparticles are not appreciably dependent on the [Ag^+^] added to the solution. However, the APB intensity shows an increase of the APB intensity as the [AgNO_3_] is increased. This dependence shows a tendency to saturation, indicating that almost all the silica has reacted.

### Influence of the Laser Wavelength on the NPs Properties

In order to evaluate the influence of the laser wavelength on the synthesis process of Ag NPs, the experiment reported in the previous paragraph was also performed using a longer laser wavelength (*λ* = 1064 nm).

**Fig. 8:**
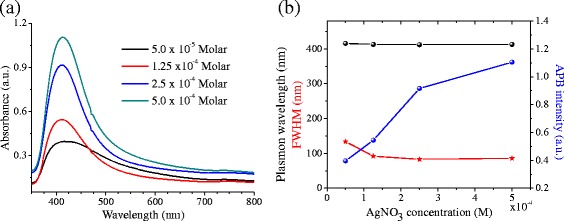
**a** Absorption spectra of Ag@Silica NPs suspensions synthesized using a laser pulse energy of 15 mJ, *t* = 10 min, *f* = 20 Hz, *λ* = 1064 nm, and different [AgNO3]. **b** Dependence of CWL, FWHM, and APB intensity on the [AgNO3]

Figure 8a shows the absorbance spectra for samples prepared with different [AgNO_3_]. Figure 8b presents the dependence of the CWL, FWHM, and APB intensity on [AgNO_3_]. These dependences follow the same trend observed in the experiments performed at the shorter wavelength (*λ* = 532 nm). The APB intensity increases with the [AgNO_3_], suggesting an increase of the [NPs]. Similarly, both the CWL and FWHM exhibit no significant dependence on the [AgNO_3_], indicating that for all the synthesized samples, the NPs are formed with similar average sizes and size distribution. However, the APB intensity values of the samples prepared at longer 1064 nm wavelength are slightly lower (0.4–1.1 a.u.) than the equivalent values of the samples prepared at 532 nm (0.97–1.4 a.u.). This indicates an overall higher [NPs] for the samples prepared with this shorter wavelength. The increase in [NPs] must be caused by an increase in the silicon amount extracted using 532 nm, which should lead to an increase in the [Ag^+^] that is reduced. Silicon absorption is higher for 532 nm than for 1064 nm; consequently, more material must be extracted from the target using the first wavelength.

**Fig. 9:**
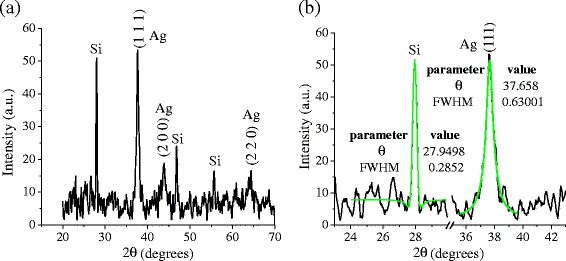
**a** XRD pattern corresponding to a colloidal suspension (Ag@Silica NPs) prepared using a laser pulse energy of 15 mJ, *λ* = 1064 nm, *f* = 20 Hz, *t* = 20 min, and a [AgNO3] of 1.25 × 10–4 M. **b** Zoom of the same pattern, which shows fitting curves used to determine de parameters of the peaks

Figure 9a shows a XRD spectrum of samples synthesized with (1.25 × 10^−4^ M) of AgNO_3_. According to the 4–0783 JCPDS card, the peaks located at 2θ = 38.17°, 44.33°, and 64.53° correspond to the reflections of the silver crystalline planes (111), (200), and (220), respectively. Using the Bragg equation, it was possible to obtain the lattice parameter (*a* = 0.413 nm), as well as the interplanar distances (*d*_111_ = 0.238 nm, *d*_200_ = 0.206 nm, and *d*_220_ = 0.145 nm), which are in agreement with the values reported in the diffraction card. To estimate the mean size of the Ag NPs only, the (111) direction was considered due to the poor fitting precision as consequence of the low signal-to-noise ratio exhibited by the reflections (200) and (220). The diameter of the Ag NPs estimated by Scherrer formula was *D*_Ag_ = (13 ± 1) nm.

Similar to the results obtained at shorter wavelength, the diffractogram of the Fig. 9a exhibits peaks located at 2θ = 27.9°, 46.8°, and 56.6° indicating the presence of crystalline silicon in the suspension, which must be ejected from the target by the shock waves produced during the ablation process. Using the Scherrer formula and the peak located 2θ = 27.9°, it was possible to estimate the size of the silicon crystallites present in the sample, *D*_Si_ = (28 ± 1) nm.

**Fig. 10:**
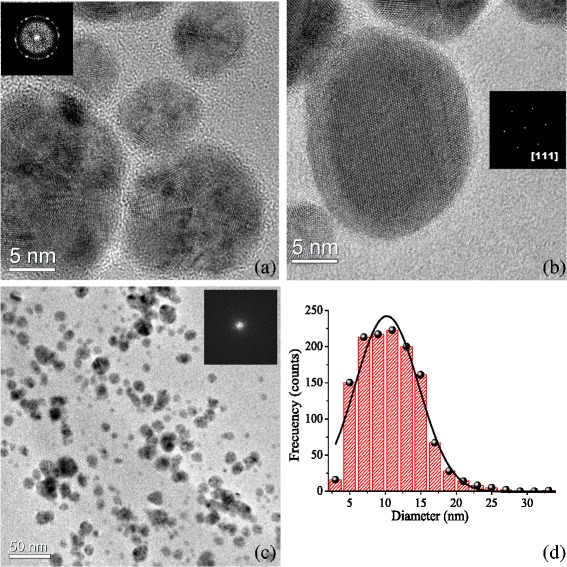
**a** and **b** HRTEM images of Ag@Silica NPs; **c** low magnification TEM image and **d** size distribution of Ag NPs synthesized using a laser pulse energy of 15 mJ, *λ* = 1064 nm, *f* = 20 Hz, *t* = 20 min, and a [AgNO3] of 1.25 × 10–4 M

Results of the HRTEM analysis of samples prepared under these conditions are presented in Fig. 10. The HRTEM images (Fig. 10a, b) show Ag@Silica NPs. The insets are the FT of the image. The circle denotes that NPs are randomly distributed over the amorphous carbon substrate. From the FT analysis, it was possible to obtain the interplanar distance of the [111] crystalline direction of the Ag FCC structure (*d*_111_ = 0.2494 nm), which agrees well with the obtained XRD diffraction results. Moreover, measuring the dimensions of 1305 NPs in the low magnification TEM images (Fig. 10c), it was possible to obtain information on the size and the size distribution of the Ag NPs. A Gaussian distribution was fitted to the obtained histogram (Fig. 10d) obtaining an average size of ${D}_{\mathrm{Ag}}^{\mathrm{ave}}=\left(10.2\pm 0.3\right)\ \mathrm{nm}$ and a standard deviation of *σ* = 4.5 nm.

## Conclusions

Silver NPs capped with silica (Ag@Silica NPs) were synthesized by means of the pulsed laser ablation of a Si solid target immersed in H_2_O and the subsequent addition of a [AgNO_3_]. Absorption spectra reveal the strong surface plasmon resonance of Ag NPs. The influence of the laser energy, laser wavelength, ablation time, and [Ag^+^] on the size and optical properties of the silver nanoparticles was investigated. Furthermore, the colloidal suspensions were studied by transmittance in the UV–VIS-NIR, X-Ray diffraction (XRD), and high-resolution transmission electron microscopy (HRTEM). The results reveal that the NPs concentration in these colloids can be controlled by the [Ag^+^], the laser pulse energy, the laser ablation time, or the laser wavelength. The silica shell coating on Ag NPs should reduce the release of silver ions, thus improving the colloidal stability and facilitating its use in biomedical applications.
